# O-41 - REVIEW OF PUBLIC HEALTH RECORDS FOR CANINE BRUCELLA CANIS NOTIFICATIONS IN 2023 IN ENGLAND

**DOI:** 10.1093/pubmed/fdag046.037

**Published:** 2026-07-09

**Authors:** Miss Katie Farnes, Miss Eva Emanuel, Dr Charlotte Robin, Dr Jennifer Taylor, Dr David Edwards, Dr Stephen Wooley, Dr Alessandro Gerada, Dr Dimple Chudasama, Dr Rachel Pudney

**Affiliations:** UK Health Security Agency; UK Health Security Agency; UK Health Security Agency; UK Health Security Agency; UK Health Security Agency; UK Health Security Agency; UK Health Security Agency; UK Health Security Agency; UK Health Security Agency

## Abstract

**Background:**

*Brucella canis* (*B. canis*) is a bacteria that causes canine brucellosis infection and can lead to zoonotic transmission from dogs to humans. *B. canis* in humans is rare, and there is limited evidence available on transmission risk and management of human contacts. The prevalence of *B. canis* in UK dogs is unknown but reports of positive dogs have increased over the last 5 years. We assessed the impact of current public health guidance and characterised the public health outcomes.

**Objectives:**

Review public health records for canine *B. canis* notified to UKHSA and summarise their outcomes.

**Methods:**

We retrospectively reviewed public health records related to all 159 canine *B. canis* notifications in England in 2023. Human contacts were categorised based on type of exposure; household, veterinary, laboratory and other. Exposures were categorised as high risk (e.g. contact with reproductive secretions), low risk (e.g. contact with saliva), and negligible risk (e.g. contact with PPE). Public health interventions and test results were summarised.

**Results:**

In total, 1,230 human close contacts were identified with 1,246 separate exposure events. There were 50 settings linked to exposure events where individual risk assessments were not recorded. Of all exposures, 74.6% were negligible-risk, followed by 12.1% low-risk, 3.8% high-risk, and 9.5% had no information available. The most common public health intervention was ‘warn and inform’ advice. No contacts were recommended antibiotic post-exposure prophylaxis. Testing was recommended for 4.5% (55/1230) contacts, however, 15 were lost to follow-up or refused testing. Of those recommended testing 72.7% were tested using serology, PCR or both, with no definitive cases of acute brucellosis diagnosed.

**Conclusions:**

Our findings support the evidence that the risk of clinically-apparent *B. canis* infection in humans exposed to infected dogs is very low. In the context of increasing canine infections, there is a need to provide clear public health guidance to avoid misinformation.

